# Comparison of three source attribution methods applied to whole genome sequencing data of monophasic and biphasic *Salmonella* Typhimurium isolates from the British Isles and Denmark

**DOI:** 10.3389/fmicb.2024.1393824

**Published:** 2024-11-14

**Authors:** Jaromir Guzinski, Mark Arnold, Tim Whiteley, Yue Tang, Virag Patel, Jahcub Trew, Eva Litrup, Tine Hald, Richard Piers Smith, Liljana Petrovska

**Affiliations:** ^1^Department of Bacteriology, Animal and Plant Health Agency, Addlestone, United Kingdom; ^2^Department of Epidemiological Sciences, Animal and Plant Health Agency, Addlestone, United Kingdom; ^3^Foodborne Infections, Department of Bacteria, Parasites and Fungi, Statens Serum Institute, Copenhagen, Denmark; ^4^Research Group for Genomic Epidemiology, National Food Institute, Technical University of Denmark, Kongens Lyngby, Denmark

**Keywords:** source attribution, monophasic and biphasic *Salmonella* Typhimurium, machine learning, random forest, Bayesian modeling, Accessory genes-Based Source Attribution, bacterial genomics

## Abstract

Methodologies for source attribution (SA) of foodborne illnesses comprise a rapidly expanding suite of techniques for estimating the most important source or sources of human infection. Recently, the increasing availability of whole genome sequencing (WGS) data for a wide range of bacterial strains has led to the development of novel SA methods. These techniques utilize the unique features of bacterial genomes adapted to different host types and hence offer increased resolution of the outputs. Comparative studies of different SA techniques reliant on WGS data are currently lacking. Here, we critically assessed and compared the outputs of three SA methods: a supervised classification random forest machine learning algorithm (RandomForest), an Accessory genes-Based Source Attribution method (AB_SA), and a Bayesian frequency matching method (Bayesian). Each technique was applied to the WGS data of a panel of 902 reservoir host and human monophasic and biphasic *Salmonella enterica* subsp. *enterica* serovar Typhimurium isolates sampled in the British Isles (BI) and Denmark from 2012 to 2016. Additionally, for RandomForest and Bayesian, we explored whether utilization of accessory genome features as model inputs improved attribution accuracy of these methods over using the core genome derived features only. Results indicated that this was the case for RandomForest, but for Bayesian the overall attribution estimates varied little regardless of the inclusion or not of the accessory genome features. All three methods attributed the vast majority of human isolates to the Pigs primary source class, which was expected given the known high relative prevalence rates in pigs, and hence routes of infection into the human population, of monophasic and biphasic *S*. Typhimurium in the BI and Denmark. The accuracy of AB_SA was lower than of RandomForest when attributing the primary source classes to the 120 animal test set isolates with known primary sources. A major advantage of both AB_SA and Bayesian was a much faster execution time as compared to RandomForest. Overall, the SA method comparison presented in this study describes the strengths and weaknesses of each of the three methods applied to attributing potential monophasic and biphasic *S*. Typhimurium animal sources to human infections that could be valuable when deciding which SA methodology would be the most applicable to foodborne disease outbreak scenarios involving monophasic and biphasic *S*. Typhimurium.

## 1 Introduction

Salmonellosis is an infection of the gastrointestinal tract that can result in diarrhea, fever, abdominal pains, and occasionally death, and is caused by the enteric bacteria of the genus *Salmonella*. It is the leading bacterial foodborne enteric disease in the US (Andino and Hanning, 2015) and second most common in the EU after campylobacteriosis (Schirone and Visciano, 2021). Worldwide, it has been estimated that there are 93.8 million cases of salmonellosis a year and 155,000 deaths (Majowicz et al., 2010). Monophasic and biphasic *Salmonella enterica* subsp. *enterica* serovar Typhimurium and *S.* Enteritidis were the most commonly reported disease-causing serovars in human patients in Europe in 2021 (European Food Safety Authority (EFSA) and European Centre for Disease Prevention and Control (ECDC), 2022). In England in 2019, monophasic and biphasic *S*. Typhimurium and *S.* Enteritidis were responsible for approximately 50% of non-typhoidal *Salmonella* infections in humans (UKHSA, 2021). Worldwide, World Health Organization (WHO) reported monophasic and biphasic *S*. Typhimurium and *S.* Enteritidis as the two most frequently reported serovars isolated in clinical practice (Fàbrega and Vila, 2013).

Monophasic and biphasic *S*. Typhimurium is a generalist serovar capable of infecting a wide range of primary hosts (Ferrari et al., 2019) that act as asymptomatic reservoirs where the pathogen lives and multiplies but does not necessarily cause a disease. The full extent of potential primary hosts of this serovar is currently unknown (Lupolova et al., 2017). Transmission of *Salmonella* leading to non-typhoidal salmonellosis could be direct, from the primary animal host, or indirect, from the food chain. Typically, *Salmonella* infections are due to the consumption of food items of animal origin (meat, diary, or eggs) from infected but asymptomatic primary animal hosts or consumption of fruits and vegetables contaminated with *Salmonella* due to the feces of an infected primary animal host (or, more rarely, human host) fouling the water used to wash the produce (Hald, 2013). Close contact with infected pets (dogs, cats, or horses) and cross-contamination at the different stages of the food production and distribution chain, such as at an abattoir, food processing plant, or during transport and distribution, are indirect sources of human infections (Stein and Chirilã, 2017). Additionally, *Salmonella* has been shown to survive in farm environments for extended periods of time and has also been isolated from animal feed and feed ingredients (Andino and Hanning, 2015).

Given that salmonellosis infections caused by monophasic and biphasic *S*. Typhimurium represent a high disease burden in both the developed and developing countries, it is vital to accurately determine the primary source of infection to discern and disrupt the routes of transmission for both sporadic cases and outbreaks. Identifying the primary source of infection or outbreak of salmonellosis, or of foodborne diseases in general, can often be difficult due to the potentially highly complex foodborne disease transmission pathways. This can be because not all infected individuals display disease symptoms, or because patients are often not able to say with any degree of certainty that consumption of a specific food product resulted in contraction of the disease (Stein and Chirilã, 2017). Source attribution (SA) is a methodology that partitions the human disease burden of a (foodborne) infection to a specific source(s), in particular the primary host, but also the consumed food (i.e., the vehicle of infection) (Pires et al., 2009). For every clinical case this approach strives to assess and quantify the importance of each of the different potential primary sources.

Source attribution is an actively developing field, and some of the recent methodologies have been extensively reviewed in Pires et al. (2009), Mughini-Gras et al. (2018), and Mughini-Gras et al. (2019), including applying these methods for human salmonellosis (Pires et al., 2014). The increasing availability of whole genome sequencing (WGS) data for a wide range of bacterial strains calls for the development of SA methods that can effectively utilize the genomic data of isolates obtained from the potential primary hosts (i.e., sources) and human salmonellosis patients as inputs into SA models (Franz et al., 2016). In a review of integrating WGS data into SA and risk assessment of foodborne bacterial pathogens, Pasquali and colleagues concluded that the application of WGS data provides improved, more specific results that can be used in decision making (Pasquali et al., 2021). Recent additions to this field include RandomForest: supervised classification machine learning algorithms, such as random forest (Lupolova et al., 2017; Zhang et al., 2019; Munck et al., 2020a; Guzinski et al., 2024); AB_SA: the Accessory genes-Based Source Attribution method, which is a multinomial logistic regression SA classifier (Guillier et al., 2020); and Bayesian: a Bayesian frequency matching method (the modified Hald method) (Arnold et al., 2021). As has recently been highlighted by Mughini-Gras et al. (2019), there is an urgent need to compare and contrast different SA approaches in order to identify methods that are best suited to specific epidemiological scenarios and can most effectively and accurately identify the source(s) of infection. Our aim in this study was to critically assess and compare the outputs of the RandomForest, AB_SA, and Bayesian source attribution methodologies by applying each of them to the same animal (i.e., primary source) and human monophasic and biphasic *S*. Typhimurium datasets.

The core genome consisting of 3,002 loci is highly conserved within distinct *Salmonella enterica* subsp. *enterica* isolates and serovars (Alikhan et al., 2018; Pearce et al., 2020). Accessory genomic elements include genetic material acquired via horizontal transfer, including extra-chromosomal plasmids, integrative and conjugative elements, replacement islands, prophages and phage-like elements, transposons, insertion sequences, and integrons (Ozer et al., 2014). Therefore, different strains are likely to harbor different accessory genomic elements (Segerman, 2012). Utilizing the accessory genome of bacterial isolates has been suggested to be a useful source of model features for SA of monophasic and biphasic *S*. Typhimurium isolates (Lupolova et al., 2017; Zhang et al., 2019). To evaluate potential improvements in the accuracy of model predictions that included the accessory genome loci as model features, SA of the analyzed monophasic and biphasic *S*. Typhimurium isolates was performed with and without the accessory genome features when applying RandomForest and Bayesian.

## 2 Materials and methods

### 2.1 Selection of strains, MLST typing, and imputation of missing data

The 904 monophasic and biphasic *S*. Typhimurium isolates analyzed in this study (Supplementary Table S1) were collected as part of Work Package 4/7 of the EU’s Horizon 2020 COMPARE research project [Collaborative Management Platform for detection and Analyses of (Re-) emerging and foodborne outbreaks in Europe, grant number 643476]. Strain selection, whole genome sequencing, quality control of the sequenced data, and multilocus sequence typing of the core genome (cgMLST) and the core and accessory genome (wgMLST) loci for this set of isolates were described in Munck et al. (2020b). WGS data of the analyzed isolates was downloaded from NCBI GenBank (Short Read Archive) using the fasterq-dump function of sratoolkit v2.9.6-1.^1^ Serotypes of the analyzed isolates were confirmed with an in-house Animal and Plant Health Agency (APHA) *in-silico* serotyping pipeline.^2^ Pipeline outputs indicated that the isolates belonged to Achtman 7-core-gene MLST sequence type 19 (ST19), ST34, ST128, ST213, ST313, ST323, ST376, ST568, ST2212, ST3228, ST3235, ST3239, ST4067 (all eBurst group 1: eBG1), and ST36 (eBG138) (Supplementary Table S1).

The dataset comprised 553 isolates sampled and sequenced in the British Isles [98.2% of the 274 British Isles (BI) animal isolates were from England and Wales] that were typed at 6,944 wgMLST loci and 351 isolates sampled and sequenced in Denmark that were typed at 6,426 wgMLST loci using BioNumerics. The combined dataset, 904 isolates in total, comprised 420 isolates from confirmed human clinical cases, including from patients with travel history outside of the BI/Denmark, and 484 animal isolates, including isolates which originated from animals not raised in the BI or Denmark (Supplementary Table S1). The 484 animal isolates belonged to the following primary source classes: 43 isolates from Broilers, 22 from Cattle, 11 from Ducks, 16 from GameBirds, 11 from Layers (laying hens), 41 from OtherMammals (companion animals such as dogs or horses), 317 from Pigs, 2 from Reptiles, 7 from Sheep, and 14 from Turkeys (Supplementary Table S1). Six-thousand-and-thirty-six wgMLST loci were common to both the BI and Danish isolate datasets such that at least a single isolate from both geographical regions was typed at a locus. Of these, 4,232 loci were typed at a minimum of 60% of the 904 isolates and were selected for further analysis. The selected locus set comprised all 3,002 cgMLST loci from the EBcgMLSTv2.0 scheme (Alikhan et al., 2018) and 1,230 out of 12,685 Applied Maths BioNumerics Salmonella MLST scheme wgMLST loci.^3^

Imputation of missing data at the 4,232 retained wgMLST loci was performed separately for the cgMLST and the accessory genome loci. Missing data for the cgMLST loci was assumed to have resulted from insufficient sequence coverage at a particular locus. The missForest R package (Stekhoven and Bühlmann, 2012), which performs non-parametric missing value imputation using random forest, was applied to impute missing data for the cgMLST loci using default parameters with exception of the *ntree* argument (number of decision trees per forest) which was set to 150. Imputation using missForest was performed separately for the 484 isolates from primary animal sources (0.4% missing data) and 420 isolates from human salmonellosis patients (0.8% missing data). For the accessory genome MLST loci the missing allele scores were all changed to “0,” which facilitated the utilization of patterns of missing data across the accessory genome dataset to differentiate between isolates from the disparate primary source classes. The most parsimonious explanation for the missing accessory genome alleles was that an isolate lacked the accessory genome element harboring a corresponding accessory genome MLST locus. The two Reptiles isolates were removed from the dataset prior to further analysis because of low numbers of samples for this primary source class.

### 2.2 Phylogeny construction

A phylogenetic tree was constructed for the 902 monophasic and biphasic *S*. Typhimurium isolates to assess the population structure and the distribution of isolates from different hosts and geographic regions of origin.

A multiple sequence alignment (MSA) for the 902 isolates was computed with snippy v4.6.0 (Seemann, 2020b) using *S*. Typhimurium eBG1, ST19, LT2 AE006468 as reference. Recombination events were removed using Gubbins v2.4.1 (Croucher et al., 2014) and subsequently SNP-sites (Page et al., 2016) was used to extract the polymorphic sites. Phylogeny of the core single nucleotide polymorphism (SNP) alignment comprising 8,147 variable sites was constructed with RAxML-NG v1.0.2 (Kozlov et al., 2019) that was run with the GTR (generalized time-reversible) nucleotide substitution model plus gamma correction, searching 100 trees (50 random and 50 parsimony-based starting trees). Branch support was assessed with 3,000 bootstrap replicates (Felsenstein’s bootstrap proportions). iTol (Letunic and Bork, 2019) was used for tree display and annotation. The tree was rooted at the biphasic *S*. Typhimurium eBG138, ST36 (SRR8820637) outgroup strain.

SnapperDB (Dallman et al., 2018) was used to assign SNP address strain level nomenclature to each of the 902 analyzed isolates (Supplementary Table S1).

### 2.3 RandomForest: supervised classification random forest machine learning algorithm

RandomForest, a supervised classification random forest machine learning algorithm (Breiman, 2001), was applied to predict the primary source classes of 482 animal isolates using cgMLST loci only (RandomForestCG model), and separately, wgMLST loci (RandomForestWG model). Prior to running the RandomForestCG and RandomForestWG models, the animal isolate dataset was split randomly 75:25 into the training and test set with 362 and 120 isolates, respectively (Supplementary Table S1). The 75:25 split ratio was maintained for each of the nine primary source classes. Identical training and test set split was used for the RandomForestCG and RandomForestWG models. The primary sources of the training set isolates were supplied to the RandomForestCG and RandomForestWG models, whereas these data were withheld for the test set which was used to assess the predictive power of the models. Random seeds used in the modeling scripts and the modeling workflow were kept constant for RandomForestCG and RandomForestWG. The tidymodels ecosystem of R programming language packages for modeling and machine learning^4^ was used for all modeling work.

To facilitate faster algorithm running times and to avoid model overfitting, the number of model features (cgMLST or wgMLST loci) was greatly reduced by filtering out the redundant features in the training set. Monomorphic cgMLST/wgMLST loci were removed using the *step_zv* function prior to running the Boruta feature selection algorithm (Kursa and Rudnicki, 2010) using the *step_boruta* function of the recipeselectors R package (Pawley, 2022).

Tuning the *mtry*, *min_n*, and *trees* RandomForestCG and RandomForestWG model hyperparameters was performed on the training set by computing 50 models each for RandomForestCG and RandomForestWG. Each model was run with a unique configuration of randomly-selected hyperparameter values. For each combination of hyperparameter values, the performance of RandomForestCG and RandomForestWG was evaluated by resampling the animal isolate training set using 10-fold cross validation. RandomForestCG and RandomForestWG models with the optimal hyperparameter configuration displayed the highest roc_auc (area under the ROC curve). The tuned RandomForestCG and RandomForestWG models were then applied to predict the primary source classes of the test set isolates. Isolates were attributed to a primary source class with the highest probability of assignment, which ranged from zero to one. A test set roc_auc value of at least 0.70 (Hosmer et al., 2013) indicated that model tuning was performed satisfactorily, and the tuned RandomForestCG and RandomForestWG models could be applied to predict the primary sources of the 420 human isolates. Prior to that step, the tuned RandomForestCG and RandomForestWG models were exposed to the entire animal isolate dataset, which enhanced the models’ predictive powers by using all available data for model training. Selection of model features for the entire animal isolate dataset was performed as described above. Ranking of model features by their importance was obtained for the tuned RandomForestCG and RandomForestWG models trained on the entire animal isolate dataset.

### 2.4 AB_SA: multinomial logistic model

Preparation of input files for the AB_SA multinomial logistic model was performed as described in Guillier et al. (2020). Genomes of the 902 monophasic and biphasic *S*. Typhimurium isolates were assembled with Shovill v.0.9.0 (Seemann, 2020a), with the *depth* and the *gsize* parameters set to 100 and 4.9M, respectively, and subsequently annotated using Prokka v1.14.6 (Seemann, 2014). The pangenome, computed with Roary v3.13.0 (Page et al., 2015), comprised a total of 13,854 genes, of which 9,921 were the accessory genes. Subsequently, Scoary v1.6.16 (Brynildsrud et al., 2016) was run as detailed in Guillier et al. (2020). The *p_value_cutoff* argument was set to 0.2, which captured the following number of enriched genes (i.e., genes for which the presence/absence patterns were associated with a specific primary source) for each primary source class: Broilers—678 genes (lowest gene-specific naive *p*-value = 2.26E-16); Cattle—256 genes (lowest gene-specific naive *p*-value = 3.40E-07); Ducks—536 genes (lowest gene-specific naive *p*-value = 1.87E-19); GameBirds—623 genes (lowest gene-specific naive *p*-value = 2.26E-10); Layers—271 genes (lowest gene-specific naive *p*-value = 1.18E-07); OtherMammals—765 genes (lowest gene-specific naive *p*-value = 5.38E-10); Pigs—1,264 genes (lowest gene-specific naive *p*-value = 1.44E-27); Sheep—413 genes (lowest gene-specific naive *p*-value = 1.92E-07); Turkey—334 genes (lowest gene-specific naive *p*-value = 1.78E-10).

The *MNLTrainTest* function of AB_SA was adapted to fit the *maxGenes* argument, which is the number of enriched genes to consider per primary source class. This was done by maximizing the accuracy of predictions and minimizing the Akaike information criterion (AIC). We selected a *maxGenes* from 1 to 12, i.e., 1 to 12 (source-enriched) genes with the lowest naive *p*-value (below 0.05 for all 108 genes) for association (presence or absence) with each of the nine primary source classes. Next, the 482 animal isolate dataset was split into 100 random training and test sets (70:30 ratio) by bootstrap resampling; each of the 100 AB_SA multinomial logistic models was trained on its respective training set and then predicted the primary source class of each of the test set isolates. The average proportion of correct predictions was taken as the accuracy of AB_SA for each *maxGenes* value (from 1 to 12). The AB_SA multinomial logistic model ran with each *maxGenes* value was then trained on the entire animal isolate dataset and the AIC was extracted from the *multinom* function of the nnet package within AB_SA. The AB_SA multinomial logistic model with the optimal number of *maxGenes* (nine) was subsequently fitted on the entire animal isolate dataset and applied to predict the primary sources of the unknown (human) samples. Each human isolate was assigned to the primary source class with the highest membership probability and secondary sources were not considered.

The AB_SA multinomial logistic model with the optimal set of predictors was also applied to predict the primary source classes of the RandomForestCG/RandomForestWG test set isolates (after being trained on the RandomForestCG/RandomForestWG training set isolates) for a direct comparison between AB_SA and RandomForestCG or RandomForestWG (only the outputs of the better performing model out of RandomForestCG or RandomForestWG were compared to AB_SA) on a set of isolates for which the primary sources were known.

### 2.5 Bayesian: frequency matching approach (the modified Hald method)

To assess the source attribution at a national level, Bayesian (the modified Hald method) was used. This is a frequency matching approach that has been used to determine source attribution for many countries (Hald et al., 2004; Glass et al., 2016; Mughini-Gras et al., 2014; Mullner et al., 2009), and has also previously been applied to WGS data (Arnold et al., 2021).

Briefly, Bayesian uses the relative frequency of occurrence of bacterial subtypes in the animal and human case data to infer the proportion of human cases that derive from each animal source. Specifically, it is assumed that human cases from source *i* and subtype *j*, *O_ij_*, follow a Poisson distribution with mean λ_ij_, which was given by:

$\lambda_{ij}=p_{ij}\;q_{i}\;a_{j}$

where *p_ij_* represents the prevalence of type *i* in food type *j*, *q_i_* represents the relative virulence of each bacterial subtype, and *a_j_* represents the relative likelihood of infection for each food source.

Bayesian was applied to the WGS data of monophasic and biphasic *S*. Typhimurium isolates sampled from primary animal hosts and human salmonellosis patients in the BI and Denmark using the following subtyping approaches, the first three of which are described in more detail in Arnold et al. (2021):

- 7-core-gene MLST
- SNP distance (SNP address) (Dallman et al., 2018)
- Hierarchical clustering based on cgMLST using EnteroBase (HCC cgMLST) (Zhou, et al., 2019)
- Hierarchical clustering based on wgMLST using a bespoke R programming language script to replicate the hierarchical clustering algorithm of EnteroBase (HCC wgMLST) (Zhou, et al., 2019)

Bayesian allows for priors to be included for each of the model parameters. In the present study, vague priors (beta distributions with both parameters equal to 1) were used for all parameters.

### 2.6 Source attribution method comparison

The degree of overlap between the DNA sequences representing two sets of genes/loci: cgMLST or wgMLST loci retained after RandomForestCG/RandomForestWG feature selection steps and the nine AB_SA source-enriched genes, was compared as described below.

Sequences of allele ‘1’ (i.e., the very first allele representing each locus) for each of the cgMLST or wgMLST loci used as RandomForestCG/RandomForestWG model features were obtained from EBcgMLSTv2.0 scheme or from Applied Maths BioNumerics Salmonella MLST scheme, respectively. Sequences of the nine source-enriched genes used by AB_SA were obtained from the *centre.fnn* Prokka output files. For each gene, DNA sequence was extracted for a single animal isolate belonging to a primary source class that the gene was associated with according to Scoary (first isolate on a list of isolates ordered by their GenBank accession numbers). For the gene that Scoary indicated was associated with the Ducks primary source class, the DNA sequence was obtained from a human isolate as that gene was absent in all isolates from Ducks in our dataset. A fasta file with the sequences of all RandomForestCG/RandomForestWG model features and a fasta file with sequences of the nine AB_SA source-enriched genes were compared for the degree of sequence overlap using the blaster R package (Tamminen et al., 2021). Blaster was run with both files as a *query* and as a *db*, and with *minIdentity* set to 0.75.

The degree of similarity between the RandomForestCG/RandomForestWG and AB_SA assigned primary source class predictions for the 120 animal test set and 420 human isolates was assessed by inspecting the primary source class each of these isolates was assigned to by the two methods. Additionally, the RandomForestCG/RandomForestWG, AB_SA, and Bayesian source attribution methods were compared for the overall proportion of human isolates assigned to the different primary sources for the entire dataset and after splitting the human samples by the “Data Owner” (Supplementary Table S1), thus either UKHSA (the BI) or SSI (Denmark).

## 3 Results

### 3.1 Phylogenetic tree and population structure of the studied isolates

**Figure 1:**
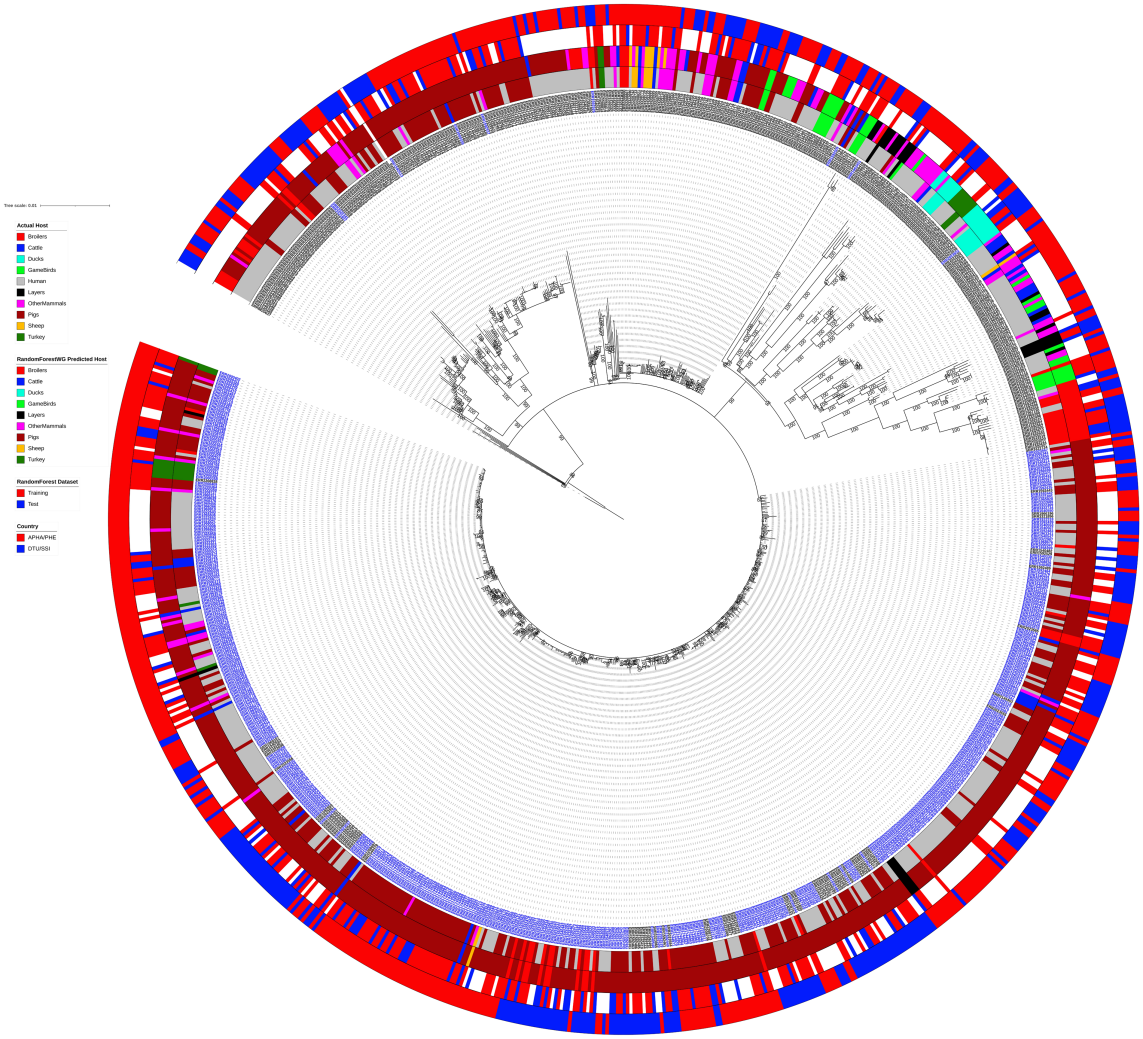
Outgroup strain (SRR8820637) rooted maximum likelihood phylogenetic tree featuring 902 animal and human biphasic *S*. Typhimurium (black isolate label) and monophasic *S*. Typhimurium (blue isolate label) isolates used for the source attribution method comparison study. The phylogeny is based on core polymorphic SNPs derived from a multisequence alignment. Innermost annotation ring specifies isolate host, second annotation ring specifies isolate assignment to a primary source class according to the RandomForestWG model, third annotation ring specifies whether an (animal) isolate was from the training or the test RandomForest dataset, and the outermost annotation ring specifies country of sampling of the analyzed isolates (APHA/UKHSA for the British Isles isolates and DTU/SSI for the Danish isolates). Bootstrap branch support values between 80 and 100% are shown on the tree.

Phylogenetic analysis grouped the 902 analyzed animal and human monophasic and biphasic *S*. Typhimurium isolates into nine main clades (Figure 1), with three of the most basal clades comprising just a couple of isolates each. The most obvious split on the tree involved separation of the monophasic *S*. Typhimurium isolates (blue isolate labels on Figure 1) from biphasic *S*. Typhimurium isolates (black isolate labels on Figure 1). The clade comprising almost exclusively the monophasic *S*. Typhimurium isolates was the largest clade of the phylogenetic tree, and, as expected, it was the clade with the shallowest branches indicating low levels of genetic diversity amongst the isolates. The maximum pairwise SNP distance amongst the 540 isolates that comprised the monophasic *S*. Typhimurium clade was 44 SNPs, whereas the maximum SNP distance between two biphasic *S*. Typhimurium isolates was 462.

Human isolates were clustered with the primary source (i.e., animal) isolates in all major clades of the tree, including the clade comprising almost exclusively the monophasic *S*. Typhimurium isolates, thus indicating that this dataset was suitable for exploring quantitative source attribution approaches as the primary sources of the investigated human isolates were likely to be present amongst the nine primary source classes. Similarly, there was no clear clustering of the isolates by their primary source class, although the large clade comprising mostly the monophasic *S*. Typhimurium isolates was made up largely of isolates sampled from Pigs and human clinical cases. Isolates sampled from the BI and Denmark were intermixed throughout the tree, as were the RandomForest training and test set isolates, thus indicating that these datasets were not biased toward isolates with specific genomic signatures (Figure 1).

### 3.2 RandomForestCG and RandomForestWG training and test set model performance

Comparison of the overall accuracy, kappa, and roc_auc model performance metrics generated by the tuned (optimal) RandomForestCG and RandomForestWG models showed that RandomForestWG correctly assigned a greater proportion of the 362 training set (88.4% RandomForestCG vs. 94.8% RandomForestWG correct assignment) and 120 test set (82.5% RandomForestCG vs. 85% RandomForestWG correct assignment) animal isolates (Table 1), indicating improved RandomForest performance with the inclusion of the accessory genome loci as model features. Detailed description of the features retained by RandomForestCG and RandomForestWG and comparison of how the two models assigned the training and the test set animal isolates to the different primary source classes is provided in the Supplementary Text and Supplementary Tables S2A,B.

**Table 1:** Number of retained features, optimal model hyperparameters, and model performance metrics for the RandomForestCG and RandomForestWG models.

	RandomForestCG	RandomForestWG
Number of model features (training set)	60	79
Best model (optimal hyperparameters)	*mtry* = 70, *trees* = 409, *min_n* = 3	*mtry* = 43, *trees* = 1,297, *min_n* = 4
Training set accuracy	0.88	0.95
Training set kappa	0.77	0.90
Test set accuracy	0.83	0.85
Test set kappa	0.66	0.70
Test set roc_auc	0.81	0.89
Number of model features (full dataset)	69	106

### 3.3 Application of RandomForestWG to predict the primary source classes of human isolates

**Figure 2:**
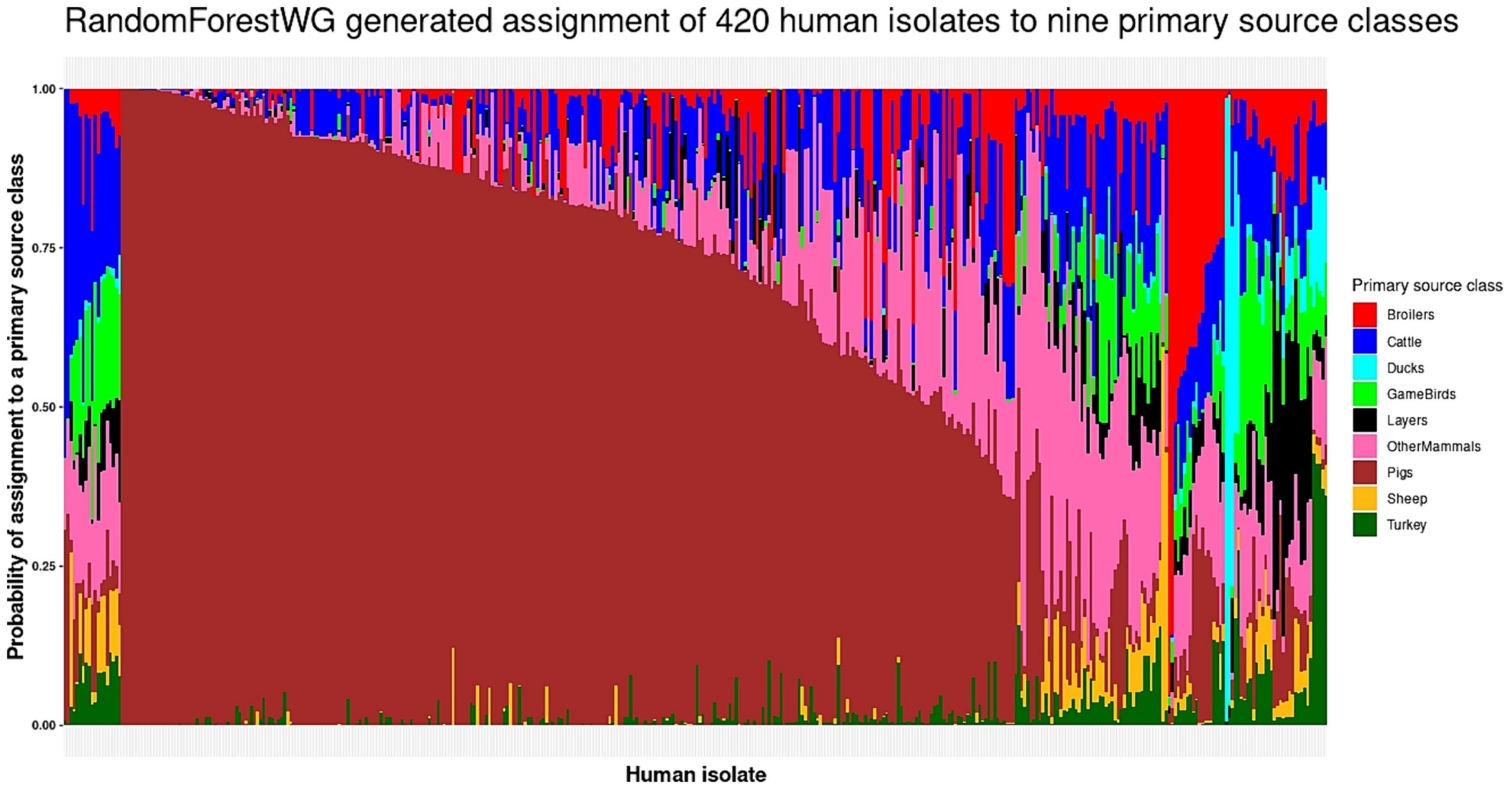
The RandomForestWG generated assignment of the 420 monophasic and biphasic *S*. Typhimurium human isolates to nine primary source classes. Each vertical bar represents a single human isolate. The color composition of each bar reflects the probability of assignment of an isolate to each of the nine primary source classes. The more uniform the color the higher the probability of assignment of an isolate to a single primary source class. The isolates are ordered by their probability of assignment to the Cattle, followed by the Pigs, OtherMammals, Sheep, Broilers, Ducks, GameBirds, Layers, and Turkey primary source classes.

**Figure 3:**
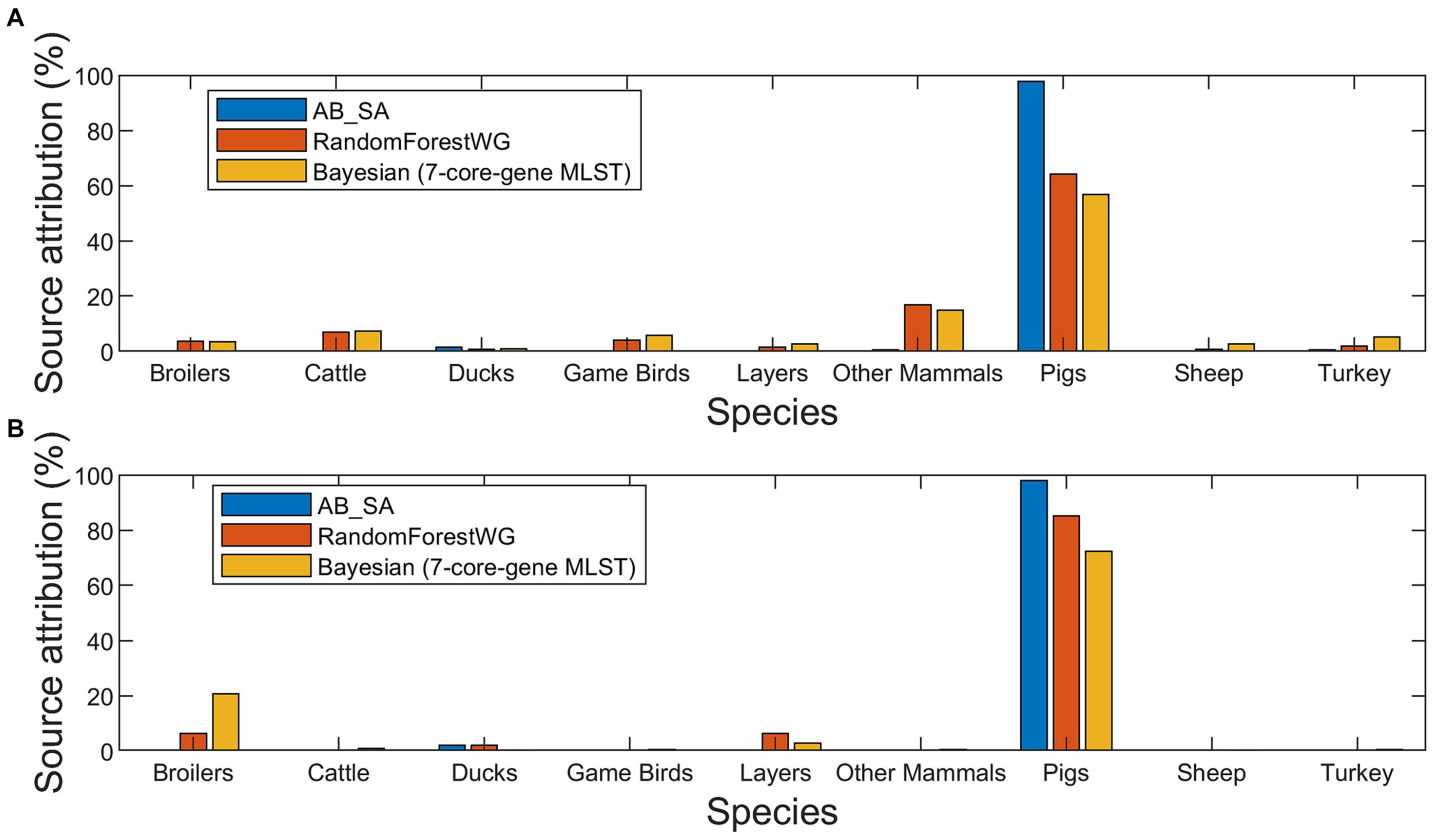
Source attribution (%) estimates of the 420 human isolates to nine primary source classes for three source attribution methods applied to monophasic and biphasic *S*. Typhimurium WGS data from the British Isles **(A)**, and from Denmark **(B)**.

The optimal RandomForestWG model (test set roc_auc = 0.89 and kappa = 0.70; Table 1) assigned 19 of the 420 human isolates to the Broilers primary source class (4.5%), 19 isolates were assigned to Cattle (4.5%), 5 to Ducks (1.2%), 11 to GameBirds (2.6%), 13 to Layers (11.2%), 299 to Pigs (71.2%), 2 to Sheep (0.5%), and 5 to Turkey (1.2%) (Figure 2 and Supplementary Table S3). The 279 BI human isolates were assigned to all nine primary source classes, with majority of the BI human isolates (64.2%) assigned to Pigs (Figure 3A and Supplementary Table S11). The 141 Danish human isolates were assigned to four primary source classes (Broilers, Ducks, Layers, Pigs), of which the vast majority (85.1%) were also assigned to Pigs (Figure 3B and Supplementary Table S12).

The highest probability of assignment of a human isolate to a primary source class was low (below 0.50) for 106 of the 121 human isolates assigned to primary sources other than Pigs (Figure 2 and Supplementary Table S3). 85.5% BI and 95% Danish human isolates were assigned to the Pigs primary source class with a probability of assignment value greater than 0.50. These patterns were likely driven by the fact that the RandomForestWG training set was dominated by the Pigs primary source class isolates (Supplementary Table S1).

### 3.4 AB_SA

When fitting *maxGenes*: the number of enriched genes considered per primary source class, the optimum solution, both in terms of maximum accuracy and minimizing the AIC, was a single gene per host species with quality of fit reducing as *maxGenes* increased (Table 2). Therefore, AB_SA was run with a total of nine genes: *srlE_2* encoding PTS system glucitol/sorbitol-specific EIIB component (soft core gene); *hypE* encoding carbamoyl dehydratase HypE (soft core gene); *thi4* encoding thiamine thiazole synthase (soft core gene); *kdgT_2* encoding 2-keto-3-deoxygluconate permease (shell (accessory) gene); *ghxP* encoding guanine/hypoxanthine permease GhxP (soft core gene); and four “groups of genes,” which all encoded hypothetical proteins (accessory genes, two of which were cloud and two were shell genes) (Supplementary Table S4).

**Table 2:** The mean overall accuracy and AIC values generated by the AB_SA multinomial logistic models ran with different numbers of genes enriched in each of the nine primary source classes (*maxGenes*).

*maxGenes*	Accuracy	AIC
**1**	**0.66**	**1208.45**
2	0.66	1268.90
3	0.64	1335.78
4	0.63	1406.63
5	0.61	1486.96
6	0.61	1555.72
7	0.60	1634.14
8	0.59	1692.10
9	0.60	1766.05
10	0.59	1869.55
11	0.59	1968.25
12	0.59	2017.01

The optimal AB_SA multinomial logistic model, with the highest mean overall accuracy and the lowest AIC (in bold) was the one with a single enriched gene per primary source class.

**Figure 4:**
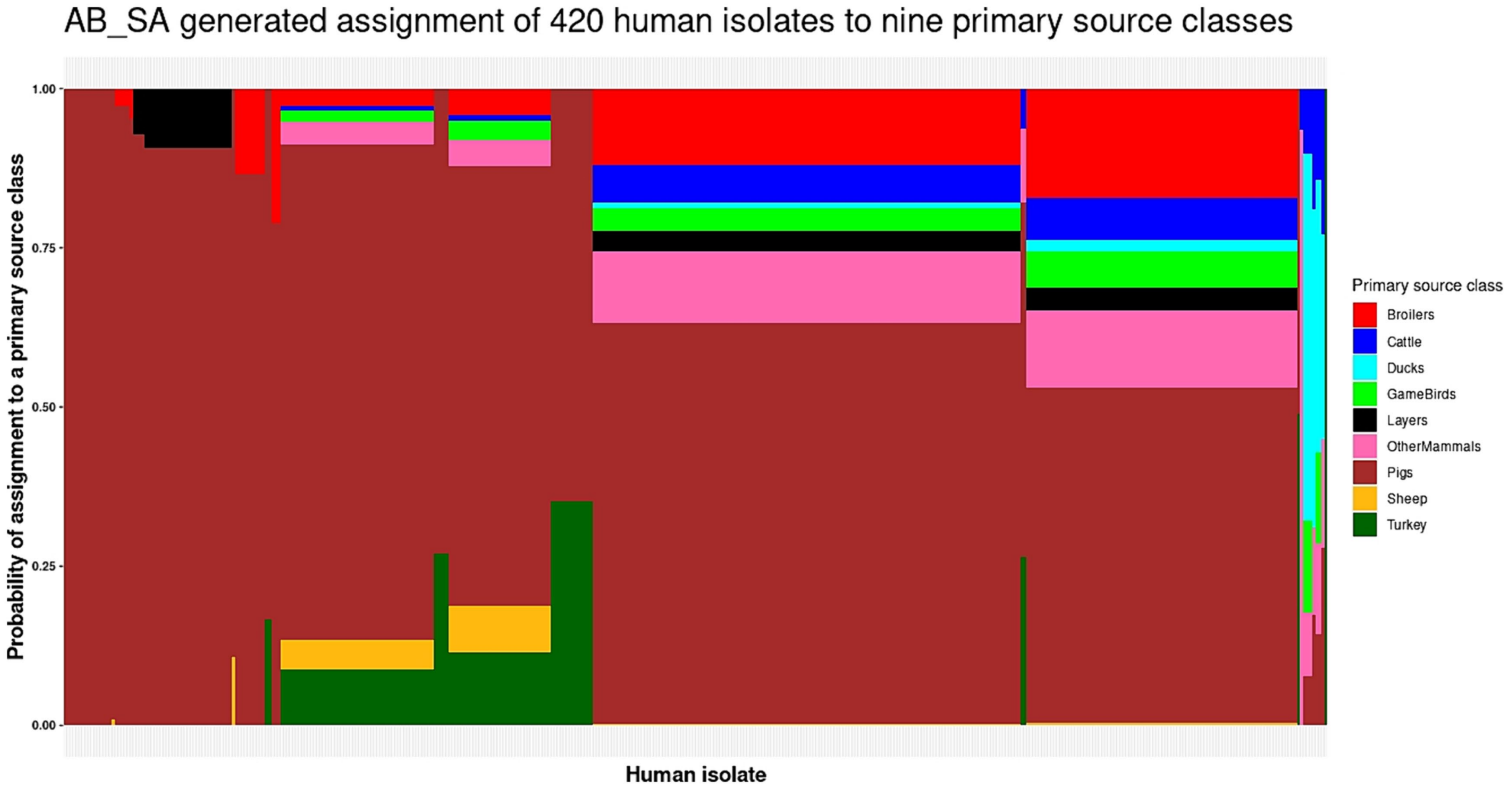
The AB_SA generated assignment of the 420 monophasic and biphasic *S*. Typhimurium human isolates to nine primary source classes. Each vertical bar represents a single human isolate. The color composition of each bar reflects the probability of assignment of an isolate to each of the nine primary source classes. The more uniform the color the higher the probability of assignment of an isolate to a single primary source class. The isolates are ordered by their probability of assignment to the Cattle, followed by the Pigs, OtherMammals, Sheep, Broilers, Ducks, GameBirds, Layers, and Turkey primary source classes.

When applying the AB_SA multinomial logistic model with a single gene per host species to predict the primary source classes of the 420 human isolates, Pigs were predicted as the primary source 98% of the time as 411 human isolates were assigned to Pigs, 7 to Ducks, 1 to OtherMammals, and 1 to Turkey (Figure 4 and Supplementary Table S5). Every one of the human isolates that AB_SA assigned to OtherMammals, Pigs, and Turkey and three of the isolates assigned to Ducks were attributed to these primary source classes with probability of assignment values exceeding 0.50. However, for four of the seven human isolates assigned to Ducks the probability of assignment values were below 0.50 and for 93 of the 411 human isolates assigned to Pigs the probability of assignment values were below 0.60, which indicated uncertainty of the AB_SA multinomial logistic model when assigning a subset of human isolates to these primary source classes (Figure 4 and Supplementary Table S5).

One issue of note is that the presence/absence of a source-enriched gene in a human clinical case isolate did not necessarily predict that the host species for which the gene was enriched was the primary source. Rather, the coefficients of the AB_SA multinomial logistic model dictated the prediction of primary source classes of the human isolates (Supplementary Table S6). For example, the *ghxP* gene was in the AB_SA multinomial logistic model as it was the top enriched gene for the Sheep primary source class. However, the coefficient for Sheep for this gene was the lowest out of all primary source classes, i.e., *ghxP* presence/absence (in the human isolates) actually reduced the probability that Sheep were predicted as the source (Supplementary Table S6). We also noticed that the intercept coefficient for the Pigs primary source was very dominant, which is why, almost regardless of the presence/absence of certain genes, Pigs were most likely to be predicted as a primary source class of the human isolates.

A couple of method modification approaches were attempted to improve the results of AB_SA but no enhancements in the accuracy were obtained. For details see the Supplementary Text.

### 3.5 Bayesian ran with different subtyping approaches

Bayesian ran with the HCC wgMLST subtyping approach was very discriminatory between samples, linking no human and animal isolates for an HCC distance of 10 or 20 for either the BI or Danish isolates (Supplementary Tables S7, S8), and linking only one human and animal isolate in the BI at an HCC distance of 50 (Supplementary Table S7). For Denmark, no isolates for GameBirds, OtherMammals, Sheep, or Turkey had the same subtype as a human isolate for any of the subtyping approaches (Supplementary Table S8), whereas for the BI most subtyping methods had at least one isolate from each animal source of the same subtype as a human isolate, except GameBirds for SNP10 (SNP address subtyping approach) and the HCC wgMLST subtyping approach at an HCC distance of 50 or less (Supplementary Table S7).

**Figure 5:**
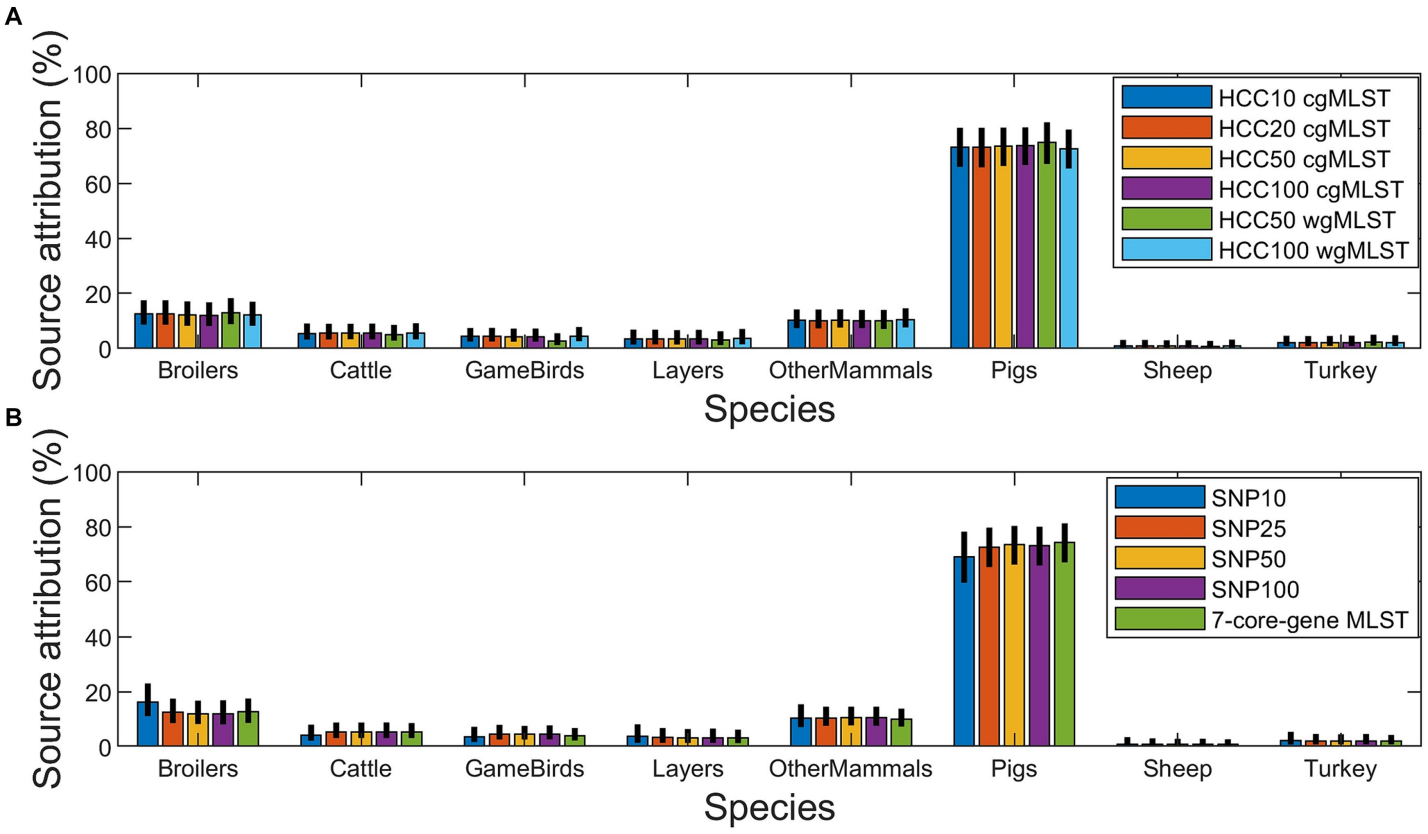
Bayesian estimates of source attribution of the 420 monophasic and biphasic *S*. Typhimurium human isolates to nine primary source classes using the HCC cgMLST and HCC wgMLST **(A)**, and SNP address and 7-core-gene MLST **(B)** subtyping approaches.

There was little overall difference in the attribution between the different subtyping approaches with most human cases being found to come from Pigs (Figure 5). However, for Denmark the second most common source was estimated to be Broilers, with very little contribution from the other sources (Figure 3B and Supplementary Table S12). For the BI, OtherMammals was the second most common source with small contributions from all the other sources (Figure 3A and Supplementary Table S11).

### 3.6 Comparison of the three source attribution methods applied in this study

Blaster searches did not identify any genes/loci that were used for SA by both RandomForestCG/RandomForestWG and AB_SA.

RandomForestWG performed better than AB_SA when predicting the primary sources of the 120 test set animal isolates for all classes except for Pigs (Supplementary Figure S1 and Supplementary Table S9). In fact, AB_SA did not correctly classify any test set isolates from the Broiler, Cattle, Ducks, GameBirds, Layers, OtherMammals, Sheep, or Turkey primary source classes, whereas RandomForestWG was 100% accurate in assigning the Ducks, Layers, and Sheep test set isolates. RandomForestWG correctly assigned 98.7% Pigs test isolates, whereas AB_SA achieved 100% accuracy for this primary source class (Supplementary Figure S1 and Supplementary Table S9).

Of the 420 human isolates, 297 (70.7%) were assigned by both RandomForestWG and AB_SA to the same primary source class (Supplementary Table S10), and this exclusively involved assignment of human isolates to the Pigs primary source class.

Comparison of how the three methods assigned the 420 human isolates revealed that the main differences were between AB_SA and the other two methods. AB_SA assigned 97.9% human isolates to the Pigs primary source class for both the BI (Figure 3A and Supplementary Table S11) and Danish isolates (Figure 3B and Supplementary Table S12). There was reasonable agreement between Bayesian and RandomForestWG, although Bayesian assigned fewer Danish human isolates to Pigs and a larger number to Broilers compared to the RandomForestWG computed assignments (Figure 3B and Supplementary Table S12).

## 4 Discussion

In this study, we compared three source attribution methods (RandomForest, AB_SA, and Bayesian) that were applied to the same dataset comprising monophasic and biphasic *S*. Typhimurium isolates collected from different primary animal hosts and patients during 2012–2016 in the BI and Denmark (Table 3). The three methods showed a different level of confidence in attributing source of human infections, based upon the methods’ different approaches in using the high-resolution molecular subtyping schemes reliant on the WGS data for attribution to primary animal sources.

**Table 3:** The advantages and disadvantages of three source attribution methods: RandomForestWG, AB_SA, and Bayesian, that were applied to the same dataset comprising monophasic and biphasic *S*. Typhimurium isolates collected from different primary animal hosts and human salmonellosis patients during 2012–2016 in the BI and Denmark.

Source attribution method	Advantages	Disadvantages
RandomForestWG	-highest accuracy of the animal isolate primary source class test set predictions -provides primary source class predictions at an individual isolate level	-model tuning requires comprehensive computing resources and/or time (approximately 1 week in the present study), although once a model has been tuned the prediction of primary sources for a set of human isolates is comparatively rapid
AB_SA	-a lot less computationally intensive than RandomForestWG -provides primary source class predictions at an individual isolate level	-lower accuracy of the animal isolate test set predictions in comparison to RandomForestWG -therefore, less effective as a predictive source attribution tool for the human isolates in the present study
Bayesian	-quickest to implement and run -useful at rapidly providing a population level estimate of source attribution	-difficult to validate the method as it only provides primary source class predictions at a population and not at an individual level -utilizes data from different subtyping approaches inefficiently as much of the data will not contribute to the final source attribution estimates

Applying RandomForest to predict the primary sources of the 420 human isolates indicated that using both the core and accessory genome loci as model features (RandomForestWG) resulted in improved model performance over a model that utilized just the core genome loci as model features (RandomForestCG) (Table 1). In the entire wgMLST set of 4,232 loci, just 29.1% belonged to the accessory genome, but in a set of loci retained after feature selection implemented prior to running RandomForestWG, the accessory genome loci comprised in excess of 40% of the retained features. Zhang et al. (2019) similarly obtained a high proportion of accessory genome loci amongst the top 50 source predicting features that were applied to predict the primary sources of biphasic *S*. Typhimurium isolates with a random forest machine learning algorithm, with 40 of the top 50 features classed as accessory genes. These results provided a strong indication that the accessory genome loci were preferentially retained after the feature selection step, possibly because accessory genes play an important role for adaptability of bacterial pathogens to different host species. It has been established that the large accessory genomes of certain *S. enterica* serovars can be host-restricted, with both gene acquisition and loss contributing to the degree of host specificity (Lupolova et al., 2017). Furthermore, previous studies have identified accessory genome features, such as genomic islands and transposons, that were suggested to encode genes which may contribute to host specificity and transmission of certain *Salmonella* serovars (Switt et al., 2012).

The optimal RandomForestWG model assigned 64.2% BI and 85.1% Danish human isolates to the Pigs primary source class (Figures 3A,B and Supplementary Tables S11, S12). Similar results were generated for a dataset that comprised largely the same Danish monophasic and biphasic *S*. Typhimurium isolates (Munck et al., 2020a). The authors of the Danish study applied machine learning algorithms ran with cgMLST loci as model features and a Bayesian frequency matching approach for which the *Salmonella* subtype was discriminated by serotyping, a multiple locus variable number tandem repeat analysis (MLVA) profile, and a phenotypic resistance profile, to perform source attribution of the analyzed human samples. These methods assigned 86.5 and 72.5% of the attributed human isolates to the Pigs primary source class, respectively (Munck et al., 2020a). Moreover, in the study described herein RandomForestWG did not assign any of the Danish human isolates to the Cattle, GameBirds, OtherMammals, Sheep, and Turkey primary source classes (Figure 3B and Supplementary Table S12). In the model training set, there was just a single isolate originating from Danish cattle and there were no Danish isolates from the GameBirds, OtherMammals, Sheep, or Turkey primary sources, which therefore precluded RandomForestWG from recognizing these primary source classes if they were of Danish origin. Therefore, the similarity of outcomes between RandomForestWG and those reported in the Danish study, and the fact that no Danish human isolates were assigned by RandomForestWG to (Danish origin) primary source classes not represented in the model training set, provided further comparative confidence in the performance of RandomForestWG.

AB_SA had a lower overall accuracy than RandomForestWG when applied to the animal isolate test set, suggesting it was less effective as a predictive source attribution tool for the human isolates in the present study (Table 3 and Supplementary Table S9). One possible reason for this is the different approach taken by AB_SA and RandomForestWG when selecting the subset of genes to be included in the model. AB_SA selected nine genes of presumed biological importance for adaptation to specific host types, however, the selection might not have corresponded with the genes which would have provided optimal performance in terms of prediction. RandomForestWG on the other hand utilized pre-selected features (MLST loci) that contributed most to the predictive power of the model. In this work, one gene per primary host (thus nine genes in total) was selected as optimal for the AB_SA multinomial logistic model as running AB_SA with higher *maxGenes* parameter values (up to 12 genes per primary source class, i.e., 108 genes in total, were tested) produced greater variability in the predicted outputs resulting in lower predictive power. A contributing factor may have been a highly imbalanced dataset on which AB_SA was trained on as it was dominated by the Pigs primary source class isolates. It is possible that if the training data were more equally weighted between the disparate host species, then the optimal *maxGenes* value would have been higher, possibly resulting in improved AB_SA predictions.

Bayesian is useful at providing a population level estimate of source attribution, which could be used to inform policy as to where to focus efforts on surveillance and control. For Bayesian, the overall attribution estimates varied little regardless of which subtyping approach was used to type the isolates (Figure 5 and Supplementary Tables S7, S8). This suggests that Bayesian, combined with 7-core-gene MLST data, can provide a time-efficient method at evaluating overall source attribution, with Bayesian being quicker to implement and run when compared to RandomForestCG/RandomForestWG or AB_SA, and 7-core-gene MLST being easier to generate than the SNP address or HCC cgMLST and HCC wgMLST subtyping approaches. One weakness of Bayesian is the difficulty in validating the method (Table 3). For the methods that infer attribution at an individual isolate level, it is possible to generate test data sets and verify the method’s performance on a subsample of the data where the source is known. This approach cannot be applied to Bayesian as this method only estimates source attribution at a population level and animal isolates, from subtypes where there are no human cases, do not contribute to the estimate at all. It also means that Bayesian utilizes the source attribution data less efficiently, as much of it will not contribute to the final source attribution estimates, and it also cannot be used to infer attribution at an isolate level unlike RandomForestCG/RandomForestWG and AB_SA. Hence, Bayesian is not applicable to individual case investigations.

The present study has a number of strengths. The large dataset of monophasic and biphasic *S*. Typhimurium cases and a substantial dataset of isolates from major food animals, sampled in the BI and Denmark and over a long time period (Supplementary Table S1), have allowed us to identify Pigs as the leading source of monophasic and biphasic *S*. Typhimurium infections in both countries. This was achieved with each of the three applied source attribution methods. Using WGS data for attribution to primary animal hosts/reservoirs is also a strength since more possible transmission routes for potential *Salmonella* (including, but not limited to, monophasic and biphasic *S*. Typhimurium) infection can be captured. One of the weaknesses of the study could be that the majority of animal isolates in the dataset belonged to the Pigs primary source (see Supplementary Text for discussion of potential influence of unbalanced dataset on RandomForestWG performance). We only had sufficient samples to estimate the relative frequency of monophasic and biphasic *S*. Typhimurium in other primary hosts, which may have influenced the estimated trend in source attribution by the different methods. However, this is consistent with the relative frequency of monophasic and biphasic *S*. Typhimurium isolations from the animal species each year in the BI (APHA, 2022) and also in Denmark.

Each of the three methods used in this study had its own advantages and disadvantages (Table 3). (1) While RandomForestWG was the most accurate in predicting the correct primary source classes of the test set animal isolates, tuning the model required close to a week of running time on a 16 core, 64 Gb RAM virtual machine. To boost the accuracy of the test set predictions, and ultimately, of the predictions of the primary sources of isolates sampled from human salmonellosis patients, it would be advantageous to add additional isolates from each of the nine primary source classes and/or additional primary source classes into the animal isolate training set. If such actions were taken, the computing resources and hence the associated costs or the time required for model tuning (there is an inverse relationship between the resources/costs and model tuning time) would be expected to increase to an even greater degree. However, once the model tuning phase has been completed, the tuned model could then be rapidly applied to predict the primary sources of a set of human isolates, as that step takes only a fraction of the time required for model tuning. (2) AB_SA uses an enrichment step to find the most relevant genes for multinomial logistic model predictions, and is orders of magnitude less computationally intensive than RandomForestWG. In this study, AB_SA had the lowest accuracy of primary host prediction when applied to the animal test set isolates. Sanchez-Pinto et al. (2018) reported that logistic regression methods require less data than other classification methods, for example random forest, to achieve stability and using a more balanced dataset might refine the model and improve statistical power and the ability to identify more relevant model predictors. (3) Predictions of the primary animal hosts by Bayesian were comparable to RandomForestWG outputs, with Bayesian also being much less computationally intensive than RandomForestWG, however with the caveat that Bayesian was only able to provide predictions at a population and not an individual isolate level. Ongoing routine surveillance of *Salmonella* (monophasic and biphasic *S*. Typhimurium, and other serovars) in food producing animals collected in a consistent sampling frame would improve the sample size and data quality for prevalence and SA estimates and allow the models underpinning each of the three tested source attribution methods to be updated regularly to monitor trends and provide timely guidance for food safety authorities as (re)-emerging *Salmonella* isolates with increased epidemiological potential change the risk associated with specific animal reservoirs.

The results generated in this study, and identification of the strengths and weaknesses of the three source attribution methods applied to a monophasic and biphasic *S*. Typhimurium isolate dataset, have substantial applicability to monophasic and biphasic *S*. Typhimurium public health responses. Being able to accurately produce SA predictions at the population level allows for the distribution of resources and pathogen control policy on the appropriate sources of infection. By identifying pigs as the main source of monophasic and biphasic *S*. Typhimurium, and thus a major source of human salmonellosis, it highlights the need to control the risk of transmission from pigs through the application of hygiene controls, especially at the slaughterhouse, to limit foodborne spread. Additionally, when identifying the likely source of infection, especially when epidemiological evidence is complex or lacking, it is important to produce a rapid result and so identifying the useful performance and speed of Bayesian was valuable. In cases where it is vital to obtain primary source predictions for human isolates at an individual rather than population level, application of RandomForest to predict the primary sources of human isolates should be strongly considered. Importantly, to rapidly achieve high accuracy human isolate primary source class predictions, the RandomForest model should be tuned and trained on a comprehensive selection of primary animal sources and isolates per primary source class.

## Data Availability

The whole genome sequencing data of the isolates analysed in this study can be obtained from GenBank. The GenBank accession numbers can be found in Supplementary Table S1.

## Footnotes

^1^ https://www.ncbi.nlm.nih.gov/books/NBK158900/

^2^ https://github.com/APHA-BAC/NextflowSerotypingPipeline

^3^ https://www.applied-maths.com/sites/default/files/extra/Release-Note-Salmonella-enterica-schema.pdf

^4^ https://www.tidymodels.org/
