# Supplementary material for: Comparison of three source attribution methods applied to whole genome sequencing data of monophasic and biphasic Salmonella Typhimurium isolates from the British Isles and Denmark
Source: Front Microbiol. 2024 Nov 14;15:1393824. doi: 10.3389/fmicb.2024.1393824 (PMC11602282; doi:10.3389/fmicb.2024.1393824)
Supplement: Supplementary file 1 [file Data_Sheet_1.zip › Data Sheet 1/Supplementary Material.docx]

Supplementary Material

Comparison of three source attribution methods applied to whole genome sequencing data of monophasic and biphasic *Salmonella* Typhimurium isolates from the British Isles and Denmark

Jaromir Guzinski^1*^, Mark Arnold^2^, Tim Whiteley^2^, Yue Tang^1^, Virag Patel^2^, Jahcub Trew^1^, Eva Litrup^3^, Tine Hald^4^, Richard Piers Smith^2^ and Liljana Petrovska^1*^

^1^Department of Bacteriology, Animal and Plant Health Agency (APHA), Addlestone, United Kingdom

^2^Department of Epidemiological Sciences, APHA, Addlestone, United Kingdom

^3^Foodborne Infections, Department of Bacteria, Parasites and Fungi, Statens Serum Institute, Copenhagen, Denmark

^4^Research Group for Genomic Epidemiology, National Food Institute, Technical University of Denmark, Kgs. Lyngby, Denmark

*** Correspondence:**Jaromir Guzinski

[Jaromir.Guzinski@apha.gov.uk](mailto:Jaromir.Guzinski@apha.gov.uk)

Liljana Petrovska

Liljana.Petrovska@ukhsa.gov.uk

# Supplementary Data

## RandomForest: comparison of model performance with (RandomForestWG) and without (RandomForestCG) accessory genome loci on training and test set animal isolates

For the RandomForestCG model, 60 core genome loci were retained after feature selection performed on the animal isolate training set and 69 core genome loci were retained after feature selection on the entire animal dataset (31 loci were common to both retained locus sets). For the RandomForestWG model, 79 loci were retained after feature selection performed on the training set, 38 (48.1%) of which were core genome and 41 (51.9%) accessory genome loci. Fifty of these 79 loci were amongst the 106 wgMLST loci (62 (58.5%) of which were the core genome loci and 44 (41.5%) the accessory genome loci) retained after feature selection on the entire animal dataset.

Forty two of the 362 training set isolates were misassigned by RandomForestCG ran with optimal hyperparameters (Table 1), the majority of which (28 isolates) were isolates from the Broilers, Cattle, Layers, OtherMammals, and Turkey primary source classes incorrectly assigned to the Pigs primary source class (Table S2A). In contrast, only 19 of the 362 training set isolates were misassigned by RandomForestWG ran with optimal hyperparameters, the majority of which (16 isolates) were incorrectly assigned to the Pigs primary source class (Table S2A). Fourteen of the 19 incorrectly assigned RandomForestWG training set isolates were also incorrectly assigned by RandomForestCG, and, in every case, it was to the same (incorrect) primary source class (Table S2A). Of the 120 test set isolates, 21 were assigned to an incorrect primary source class by RandomForestCG, which included misassignment of ten isolates from the Broilers, Cattle, and OtherMammals primary source classes to Pigs (Table S2B). Eighteen test set isolates, 17 of which were also misassigned by RandomForestCG, were assigned to an incorrect primary source class by RandomForestWG. These misassignments were mostly due to the incorrect assignment of 12 Broilers, Cattle, OtherMammals, and Turkey isolates to the Pigs primary source class (Table S2B).

Of the 47 training set isolates that were incorrectly assigned by the RandomForestCG and RandomForestWG models, there were 14 isolates misassigned by both models, 28 isolates misassigned by RandomForestCG only and just five isolates misassigned by RandomForestWG only (Table S2A). Of the 22 test set isolates that were incorrectly assigned by RandomForestCG and RandomForestWG, 17 isolates were incorrectly assigned by both models, four isolates were incorrectly assigned by RandomForestCG only and just a single isolate was incorrectly assigned by RandomForestWG only (Table S2B). Thus, overall, there were fewer animal training and test isolates assigned to incorrect primary source classes by RandomForestWG compared to the number of incorrect assignments of the animal training and test isolates by RandomForestCG, providing support for improved model performance if the accessory genome loci were included in the set of model features.

## RandomForest: potential influence of the majority class on RandomForestWG model performance

The vast majority of the training and test set animal isolates that were assigned to an incorrect primary source class by RandomForestWG involved the model incorrectly assigning isolates from classes other than Pigs to the Pigs primary source class. This outcome was likely driven by the fact that the majority (65.7%) of the training set isolates (and of the entire set of animal isolates - 65.8%) belonged to the Pigs primary source class and hence this was the primary source class that the model was exposed to most frequently during the training phase. Other studies that implemented supervised classification machine learning algorithms to categorize bacterial pathogens based on unbalanced training sets concluded that upsampling of the samples from the classes least well represented in the training set improved the performance of the models (Njage et al. 2019, Munck et al. 2020). Here, upsampling of the training set isolates from classes other than Pigs to the level of that primary source class was explored, although only preliminarily, as the overall aim of the study was to compare performance of three source attribution methods applied to an exact same dataset. The preliminary upsampling of the training set isolates indicated that the RandomForestWG model applied to an upsampled dataset overall performed worse than the RandomForestWG model applied to the original dataset. Analysing the outputs of RandomForestWG ran on an upsampled dataset revealed that there were only very few incorrect assignments of isolates from other primary source classes to the Pigs primary source, but rather, the incorrect assignments were dominated by the incorrect assignment of the Pigs isolates to primary source classes other than Pigs. More thorough exploration of how the upsampling of the training set isolates affected RandomForestWG performance and also exploration of other methods/algorithms to achieve a balanced training set likely deserves further exploration and a separate study.

## AB_SA: attempted improvements

In this paper we followed AB_SA exactly as it is described in Guillier et al., (2020). However, we also attempted to improve the method by modifying the default approach. Specifically, we looked at two areas: running AB_SA with three primary source classes only - Pigs and the pooled Poultry and Ruminant classes and ordering the genes enriched within each primary source class by criteria other than the naive p-value (running AB_SA on the entire 482 animal isolate dataset and nine distinct primary source classes).

For the first AB_SA improvement attempt, Roary v3.13.0 and Scoary v1.6.16 were run on the 3-class animal isolate dataset which was subsequently split into 100 random training and test sets (70:30 ratio) prior to running AB_SA. The AB_SA multinomial logistic models (one AB_SA multinomial logistic model for each training:test split) were then trained on the training set and tasked with predicting the primary source class of each of the test set isolates. One potential caveat to this split is that as all of the available animal data were used for running Roary and Scoary, there was a chance of data leakage, i.e., the selection of the source-enriched genes was potentially not specific to the test set only but influenced by the entire 3-class animal isolate dataset and hence also the training set.

This was accounted for in the second attempt at improving AB_SA as the animal isolate dataset was split 75:25 into a training and test set, respectively, replicating the data preparation steps taken prior to running the RandomForestCG/RandomForestWG models. Roary and then Scoary were run just on the training set, which ensured that there was no data leakage. Subsequently, AB_SA was run on the test set after selection of the source-enriched genes in training set only. Accuracy of the AB_SA multinomial logistic model reflected the percentage of the correctly assigned test set isolates.

## Running AB_SA with three primary source classes

In their original paper, Guiller et al., (2020) used only three primary source classes: Pigs, Poultry, and Ruminants when running AB_SA. We followed this approach by combining the Broilers, Ducks, Layers, and Turkey primary source classes isolates into Poultry (n=79) and Cattle and Sheep primary source classes isolates into Ruminants (n=29). All Pigs primary source class isolates remained in the dataset, whereas the GameBirds and OtherMammals isolates were removed.

*maxGenes* indicated that the optimal number of enriched genes per primary source class was two and the highest accuracy of prediction was 74.6%. The percentage of the Pigs primary source class isolates in the test set of the 3-class animal isolate dataset was 74.2%. Therefore, how the isolates were attributed to the primary source classes by AB_SA was a very slight improvement on a random assignment of the isolates to one of three primary source classes and hence this approach could be investigated further.

## AB_SA: ordering source-enriched genes by sensitivity/specificity

Pigs primary source class isolates comprised 65.8% of the test set. Selection of enriched genes for each primary source class based on ordering of the Scoary outputs by the naive p-value (as per Guiller et al., (2020)), by sensitivity, or by specificity produced accuracies of 65.8%, 53%, and 65%, respectively. Therefore, there were no improvements in how well AB_SA assigned the test isolates to their correct primary source classes if the selection of the source-enriched genes was based on criteria other than what is proposed in Guiller et al., (2020).

# Supplementary Figures and Tables

**Figure S1** Percentage of the 120 monophasic and biphasic *S*. Typhimurium test set animal isolates correctly assigned to their actual primary source class shown for nine primary source classes for RandomForestWG and AB_SA.

**Table S1** Metadata, including GenBank accession number, the WGS data owner, serovar, 7-core-gene MLST sequence type (ST), SNP address, country of origin (animal isolates) or patient travel history (human isolates), year of isolation, primary source class, and whether an isolate was part of the training or test set of RandomForest for the 902 monophasic and biphasic *S*. Typhimurium isolates analysed in this study. Note that the two British Isles Reptile isolates that were removed from the monophasic and biphasic *S*. Typhimurium animal isolate dataset prior to the analysis are not included in the table.

**Table S2** Assignment of the 362 monophasic and biphasic *S*. Typhimurium training set animal isolates (A) and the 120 monophasic and biphasic *S*. Typhimurium test set animal isolates (B) by the RandomForestCG and RandomForestWG models. Isolates that were assigned by the models to primary source classes other than the actual primary source class are highlighted in yellow.

**Table S3** The RandomForestWG generated probability of assignment values to each of the nine primary source classes for each of the 420 monophasic and biphasic *S*. Typhimurium human isolates.

**Table S4** Prokka gene annotation for the nine AB_SA source-enriched genes or “groups of genes” (a single gene per host species) and gene frequency in the analysed animal isolate dataset: soft core genes were present in 95% to 99% of strains, shell (accessory) genes in 15% to 95%, and cloud (accessory) genes in 0% to 15%.

**Table S5** The AB_SA generated probability of assignment values to each of the nine primary source classes for each of the 420 monophasic and biphasic *S*. Typhimurium human isolates.

**Table S6** The intercept and the host specific coefficients of the AB_SA multinomial logistic model.

**Table S7** The number of the monophasic and biphasic *S*. Typhimurium animal isolates for different subtyping approaches of Bayesian whose type corresponded to that also found in humans’ data from the British Isles.

**Table S8** The number of the monophasic and biphasic *S*. Typhimurium animal isolates for different subtyping approaches of Bayesian whose type corresponded to that also found in humans’ data from Denmark.

**Table S9** Comparison of how RandomForestWG and AB_SA assigned the 120 monophasic and biphasic *S*. Typhimurium test set animal isolates and estimation of the proportion of the correctly assigned isolates by each method for the nine primary source classes.

**Table S10** Comparison of how RandomForestWG and AB_SA assigned the 420 monophasic and biphasic *S*. Typhimurium human isolates to nine primary source classes.

**Table S11** Source attribution (%) estimates for 279 of 420 human isolates to nine primary source classes for three source attribution methods applied to monophasic and biphasic *S*. Typhimurium WGS data from the British Isles.

**Table S12** Source attribution (%) estimates for 141 of 420 human isolates to nine primary source classes for three source attribution methods applied to monophasic and biphasic *S*. Typhimurium WGS data from Denmark.

References:

Guillier, L., Gourmelon, M., Lozach, S., Cadel-Six, S., Vignaud, M. L., Munck, N., et al. (2020). AB_SA: Accessory genes-Based Source Attribution - tracing the source of Salmonella enterica Typhimurium environmental strains. *Microb. Genom.* 6:mgen000366.

Munck, N., Njage, P. M. K., Leekitcharoenphon, P., Litrup, E., Hald, T. (2020). Application of whole‐genome sequences and machine learning in source attribution of Salmonella Typhimurium. *Risk Anal.* 40, 1693-1705. doi: 10.1111/risa.13510

Njage, P. M. K., Leekitcharoenphon, P., & Hald, T. (2019). Improving hazard characterization in microbial risk assessment using next generation sequencing data and machine learning: predicting clinical outcomes in shigatoxigenic Escherichia coli. *Int. J. Food Microbiol.* 292, 72-82. doi: 10.1016/j.ijfoodmicro.2018.11.016
