# Supplementary figures and images for: Comparison of three source attribution methods applied to whole genome sequencing data of monophasic and biphasic Salmonella Typhimurium isolates from the British Isles and Denmark
Source: Front Microbiol. 2024 Nov 14;15:1393824. doi: 10.3389/fmicb.2024.1393824 (PMC11602282; doi:10.3389/fmicb.2024.1393824)

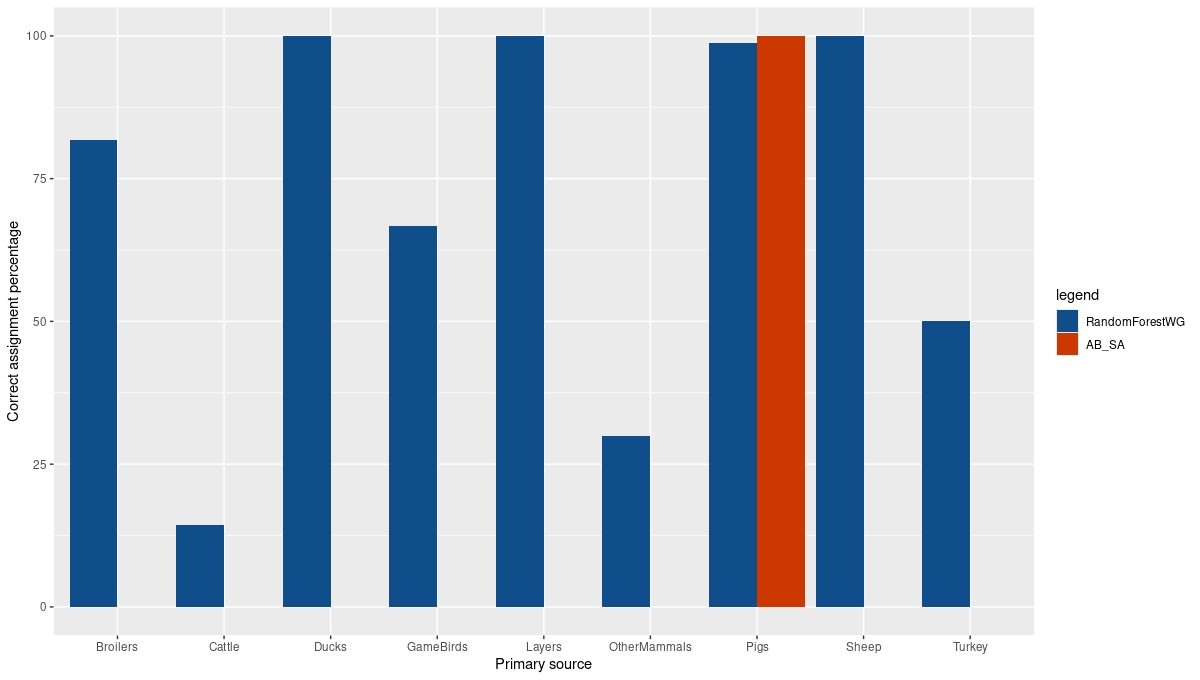

Supplement: Supplementary file 1 [file Data_Sheet_1.zip › Data Sheet 1/Supplementary Figure 1.jpeg]
